# Magnetic Nanoparticles as a Component of Peptide-Based DNA Delivery System for Suicide Gene Therapy of Uterine Leiomyoma

**DOI:** 10.3390/bioengineering9030112

**Published:** 2022-03-08

**Authors:** Sofia Shtykalova, Anna Egorova, Marianna Maretina, Vladislav Baranov, Anton Kiselev

**Affiliations:** Department of Genomic Medicine, D.O. Ott Research Institute of Obstetrics, Gynecology and Reproductology, Mendeleevskaya Line 3, 199034 Saint-Petersburg, Russia; sofia.shtykalova@gmail.com (S.S.); egorova_anna@yahoo.com (A.E.); marianna0204@gmail.com (M.M.); baranov@vb2475.spb.edu (V.B.)

**Keywords:** DNA delivery, peptide-based carriers, gene therapy, magnetic nanoparticles, thymidine kinase, uterine leiomyoma, integrins

## Abstract

Suicidegene therapy is considered a promising approach for the treatment of uterine leiomyoma (UL), a benign tumor in women characterized by precise localization. In this study, we investigate the efficiency of αvβ3 integrin-targeted arginine-rich peptide carrier R6p-cRGD electrostatically bound to magnetic nanoparticles (MNPs) for targeted DNA delivery into the UL cells. The physico–chemical and cytotoxic properties, transfection efficiency, and specificity of R6p-cRGD/DNA/MNPs polyplexes were evaluated. The addition of MNPs resulted in a decrease in the time needed for successful transfection with simultaneous increase in efficiency. We revealed a therapeutic effect on primary UL cells after delivery of plasmid encoding the herpes simplex virus type 1 (HSV-1) thymidine kinase gene. Treatment with ganciclovir resulted in 20% efficiency of suicide gene therapy in UL cells transfected with the pPTK-1 plasmid. Based on these results, we conclude that the use of cationic peptide carriers with MNPs can be promising for the development of modular non-viral carriers for suicide gene delivery to UL cells.

## 1. Introduction

Uterine leiomyomas (ULs) occupy the second place in the structure of gynaecological pathology and are the most common tumor of the female reproductive tract [1]. According to statistical data, the frequency of ULs is from 35 to 70% among reproductive-aged women [2]. Despite being benign, these tumors can cause severe symptoms such as excessive uterine bleeding, pelvic pain, menorrhagia, infertility, and pregnancy loss [3]. These clinical complications affect women’s health and quality of life and are also the most common reasons for hysterectomy [4]. In addition to hysterectomy, various minimally invasive surgeries are used, e.g., laparoscopy and minimally invasive myomectomy, but the re-appearance of new tumors remains a significant problem. In addition, drug treatments, such as the use of GnRH antagonists and aromatase inhibitors, have been developed; however, hypoestrogenic side-effects, such as bone loss, occur [5]. Thus, the development of new approaches to treatment is still required. The absence of metastasis, the precise localization of leiomyoma nodes detected by ultrasound, and the availability of various endoscopic techniques make UL an ideal target for gene therapy in situ.

Gene therapy is a type of therapy that involves various strategies to deliver nucleic acids and their derivatives into the cell in achieving therapeutic benefits. It has proven to be a promising therapeutic strategy that circumvents the limitations of pharmacological treatments and can be applied to both inherited and acquired diseases. Significant advancements in development of gene therapy have recently been made and several new drugs have been approved by the FDA [6]. Since UL are hormone-dependent tumors, gene therapy focused on influencing the metabolism of estrogen and progesterone, seems to be the most promising. Research devoted to the delivery of the dominant-negative estrogen receptor (DNER) gene into UL cells has demonstrated a significant decrease in tumor size and development [7]. DNER is a form of estrogen receptor (ER) that is unable to activate transcription. In addition, DNER molecules form dimers with native ER and inhibit their activity [8].

Another approach to UL gene therapy that is actively studied is suicide gene therapy. Suicide gene therapy is a frequently applied method based on the delivery of enzyme genes, usually bacterial or viral, which transforms non-toxic pro-drugs into a toxic form [9]. The most common system used in suicide gene therapy is the herpes simplex virus 1 thymidine kinase gene (HSV1-TK) transfer followed by delivery of a guanosine analogue, aciclovir (ACV) or ganciclovir (GCV) [10]. The delivery of the HSV1-TK gene with subsequent ACV or GCV treatment leads to decrease inthe proliferative activity of UL cells and the size of tumors [11].

The ongoing problem of UL gene therapy is the development of efficient carriers for genetic constructs’ delivery into the fibroids tissue. Most of the previously done research was held using viral vectors, in particular, Ad vectors and their different modifications [12,13]. However, despite the high efficacy of transduction, usage of viral vectors in clinical practice is limited due to their immunogenicity and toxicity. Non-viral carrier systems are promising for gene delivery. They are non-toxic and biodegradable, and their application is not limited by severe immunogenicity and inflammation problems. Besides, gene delivery into UL is challenging because of dense cell arrangement and excessive extracellular matrix (ECM), which is one of the key features of the disease [14]. In order to overcome these barriers and successfully deliver therapeutic genes into deep layers of the tumor, vectors are modified with ligands and additional components, such as magnetic nanoparticles [15].

Biocompatible magnetic nanoparticles have been developed to increase the efficiency of the gene delivery, especially in the field of non-viral delivery. Presently the efficiency of the method often referred to as “magnetofection” has been confirmed in various cell cultures. The fundamental principle of magnetofection is the binding of transfection reagents or viruses to magnetic nanoparticles (MNPs), forming molecular polyplexes.

Covalent or non-covalent binding of magnetic nanoparticles with genetic vectors makes it possible to concentrate polyplexes, thereby allowing a reduction in the dose of the vector used. This is crucial in the case of the use of viral vectors, unsuitable doses of which can cause an immune response [16]. The action of an external magnetic field allows targeting and accelerating the delivery of genetic constructs, facilitating cellular absorption [17]. Association of MNPs with non-viral carriers allows increasing the efficiency of delivery significantly. Thus, binding of DNA-polyplexes formed with cell-penetrated peptides to magnetic nanoparticles showed high efficiency of gene delivery into the cells, while reducing the time required to achieve cell membranes [18]. Magnetofection with adenoviral delivery was successfully applied in research devoted to uterine fibroids and fibroid-associated tumor stem cells. However, no studies on magnetically enhanced non-viral transfer to UL cells have been carried out. Further studies of this approach can lead to the prevention of the re-occurrence of the tumors [19].

In previous reports, we demonstrated that Arg-rich peptide carriers modified with αvβ3 ligand can effectively deliver DNA into the αvβ3-expressing tumor cells, including UL cells [20,21].This work aimed to explore whether the use of MNPs together with R6p-cRGD-polyplexes can improve the delivery rate and become a promising tool for the development of UL gene therapy.

## 2. Materials and Methods

### 2.1. Cell Lines

The human pancreatic (PANC-1) cell line was obtained from the Cell Collection of the Institute of Cytology RAS (Saint-Petersburg, Russia). The PANC-1 cells were cultured in Dulbecco’s Modified Eagle Media (DMEM; Biolot) with the addition of 10% FBS and 0.3% gentamicin. Primary UL cells were isolated from UL nodes after myomectomy at the D.O. Ott Research Institute of Obstetrics, Gynecology and Reproductology (Saint-Petersburg, Russia), as previously reported [22,23]. The UL cells were cultured in AmnioMAX C-100 Complete Medium (Thermo Fisher Scientific, Carlsbad, CA, USA) and transfection experiments were held in DMEM-F12 (Thermo Fisher Scientific) supplemented with 10% FBS (Thermo Fisher Scientific) and 0.3% gentamicin.

### 2.2. Peptide Carrier, Expression Plasmids and Magnetic Nanoparticles

Monomers of peptide carrier R6p-cRGD were synthesized in NPF Verta, LLC (Saint-Petersburg, Russia) and stored as a dry powder at −20 °C. R6p-cRGD carrier was obtained by polycondensation reaction, as described previously [21]. As a control carrier, branched polyethyleneimine (PEI) with molecular weight of 25 kDa (Sigma Aldrich, St. Louis, MO, USA) was used. Magnetic nanoparticles combiMAG were purchased from Chemicell (Berlin, Germany).

The pCMV-lacZ plasmid carrying the β-galactosidase gene was giftedby Professor B. Sholte (Erasmus University, Rotterdam, The Netherlands). The pPTK-1 plasmid encoding the HSV1 herpes virus thymidine kinase gene was kindly provided by Dr S.V.Orlov (Institute of Experimental Medicine, Saint-Petersburg, Russia). The plasmids were purified using a Qiagen Plasmid Giga kit (Qiagen, Hilden, Germany) under endotoxin-free conditions, diluted in water to 0.5–1 mg/mL, and stored at −20 °C.

### 2.3. Formation of R6p-cRGD/DNA/MNPs Polyplexes

Nucleopeptide polyplexes were formed by electrostatic binding of positively charged carrier R6p-cRGD and negatively charged expression plasmids (pCMV-lacZ, pPTK-1). To form nucleopeptide polyplexes, plasmid DNA and peptide carrier R6p-cRGD were first dissolved separately in HBM buffer (0.27 M mannitol, 5 мM HEPES, NaOH, pH 7.5) and water, respectively, and then mixed at a 1:1 volume ratio. Nucleopeptide polyplexes were allowed to self-assemble for 30 min at room temperature. Then, combiMAG MNPs were added to the DNA/peptide complex suspension according to the manufacturer’s recommendations and the suspension was incubated for 15 min at room temperature. DNA binding was evaluated by ethidium bromide displacement [24].

### 2.4. DNA Binding and DNAse I Protection Assay

The stability of nucleopeptide polyplexes of DNA with R6p-cRGD after addition of magnetic nanoparticles was analyzed using the ethidium bromide (EtBr) fluorescence quenching method of Wallac 1420D scanning multilabel counter (PerkinElmer Wallac Oy, Turku, Finland) at 590 nm emission (540 nm excitation) with an exposure time of 5 s. The EtBr displacement assay was performed in 96-well flat-bottom plates (Medpolymer, Saint-Petersburg, Russia). The efficiency of EtBr displacement was calculated as (F − Ff)/(Fb − Ff), where Ff and Fb are the EtBr fluorescence values in the absence and presence of DNA, respectively [24]. For each DNA/carrier ratio, the intensity of fluorescence (F) was measured three times.

The DNAse I protection assay requires 10 μL of the peptide/DNA/MNPs polyplexes prepared with different amounts of MNPs and incubated with 0.5 units of DNase I (Ambion, Austin, TX, USA) for 30 min at 37 °C followed by 2 min of DNAse I activation, as described previously [22]. DNA was released from polyplexes with overnight 0.1% trypsin incubation at 37 °C. The DNA integrity was analyzed by 1% agarose gel electrophoresis.

### 2.5. Relaxation of Carrier/DNA Polyplexes by Dextran-Sulfate and DTT Destabilization

Dextran-sulfate (DS; Sigma, St. Louis, MO, USA) was added to the prepared polyplexes with MNPs at three-fold charge excess relative to the peptide. At 0 min and after 1 h, 6 h, and 24 h of incubation, EtBr fluorescence was measured on Wallac 1420D scanning multilabel counter and dye displacement was evaluated.

For study of DTT destabilization, the peptide/DNA/MNPs polyplexes were prepared with the addition of 1× SYBR-Green (Amresco, Ohio, OH, USA), followed by incubation with 200 mM dithiothreitol (DTT) for 1 h at 37 °C. The fluorescence was measured, and SYBR-Green displacement calculated as (F − Ff)/(Fb − Ff), where Ff and Fb are the fluorescence intensities of SYBR-Green in the absence and presence of DNA.

### 2.6. Size and z-Potential Measurement of Peptide/DNA/MNPs Complexes

The size and zeta potential of the polyplexes with MNPs were determined using dynamic light scattering and microelectrophoresis, respectively. The measurements were performed using zetasizer NANO ZS (Malvern instruments, Malvern, UK) three times independently.

### 2.7. Transmission Electronic Microscopy

Micrographs of the R6p-cRGD/DNA polyplexes at 8/1 and 12/1 N/P charge ratios bound to MNPs in DNA:MNPs quantity ratio of 1:0.5 were obtained using a transmission electron microscope Jeol JEM-1400 (JEOL Ltd., Tokyo, Japan). A method of negative staining with a 1% aqueous solution of uranyl acetate was used to obtain electron micrographs.

### 2.8. Transfection of PANC-1 Cells with DNA/Peptide Polyplexes with MNPs

PANC-1 cells were seeded in 48-well cultural plates at a density of 65,000 cells per well in 500 μL of the standard culturing medium, grown for 24 h, and washed with DMEM, with the subsequent addition of 500 μL of serum-free DMEM into each well. R6p-cRGD/DNA/MNPs polyplexes in HBM buffer were added at a rate of 2 μg of DNA per well. The plate was incubated on a magnet (IBA GmbH, Gottingen, Germany) in a temperature-controlled chamber at 37 °C with 5% CO_2_ for 10 min and washed with DMEM. Then, 500 μL of the standard cultural medium was applied to each well, the plate incubated at 37 °C with 5% CO_2_ for 48 h, and reporter gene expression analyzed.

### 2.9. Cytotoxicity Assay

The cytotoxicity of R6p-cRGD/DNA/MNPs polyplexes was rated on PANC-1 cells using Alamar Blue reagent (BioSources International, San Diego, CA, USA) after 10, 20, and 30 min of incubation with it, as described previously [25]. Fluorescence was measured on Wallac 1420D scanning multilabel counter (excitation 544 nm, emission 590 nm) and the relative fluorescence intensity was calculated.

### 2.10. Suicide Gene Therapy of Primary UL Cells

Primary leiomyoma cells were seeded onto 96-well plates at a density of 20,000 cells per well in 100 μL of AmnioMAX C-100 (Thermo Fisher Scientific) and cultured for 24 h. Transfections were performed in a serum-free DMEM-F12 with 0.35 µg of DNA per well. The plates were incubated for 30 min on the magnet and the medium was changed to DMEM-F12 with FBS (Thermo Fisher Scientific) for the next 24 h. After that, the medium was changed to DMEM-F12 with FBS containing ganciclovir (5 μg per well) and the plate was left for 96 h.

For proliferation activity measurement 96 h later, the medium was replaced with a fresh one containing 10% Alamar Blue solution, and the plate was incubated for another 2 h. Fluorescence intensity was measured using a Wallac 1420D scanning multilabel counter at a wavelength of 530/590 nm. The relative fluorescence was calculated as (F − Ff)/(Fb − Ff), where Fb and Ff are the fluorescence intensities in the untreated control and without cells, respectively.

Photographs of cells were obtained by microscope MIBR (LOMO, Saint-Petersburg, Russia) at 200× magnification.

To count living cells, the Trypan blue dye staining method was used. After 96 h of incubation, the cells were harvested with Trypsin-EDTA (Thermo Fisher Scientific, Carlsbad, CA, USA) 0.25%, followed by the addition of 0.4% Trypan blue solution (Sigma-Aldrich, Munich, Germany) at a 1:1 volume ratio. The unstained cells were counted using a hemocytometer (MiniMedProm, Dyatkovo, Russia). Effects ofsuicide gene therapy were evaluated by normalizing live cell numbers in the treated wells to those in the untreated ones.

### 2.11. Data Analysis and Statistical Comparisons

Statistical analyses were performed using Prism 6 (Graphpad, La Jolla, CA, USA). Measurements were taken from distinct samples, and no data were excluded. The distribution of values in all experiments was evaluated by the Shapiro–Wilk normality test. In case the values were not normally distributed the nonparametric test (Kruskal–Wallis with Dunn’s multiple comparison test) was used for statistical comparisons. If the datasets were normally distributed the parametric test (ANOVA with Sidak’s multiple comparison test) was used for statistical comparisons. All the statistical tests were two-sided and conducted at 5% statistical significance.

## 3. Results

### 3.1. Evaluation of R6p-cRGD/DNA Polyplexes Stability and Protecting Properties after Non-Covalent Bindingwith MNPs

**Figure 1:**
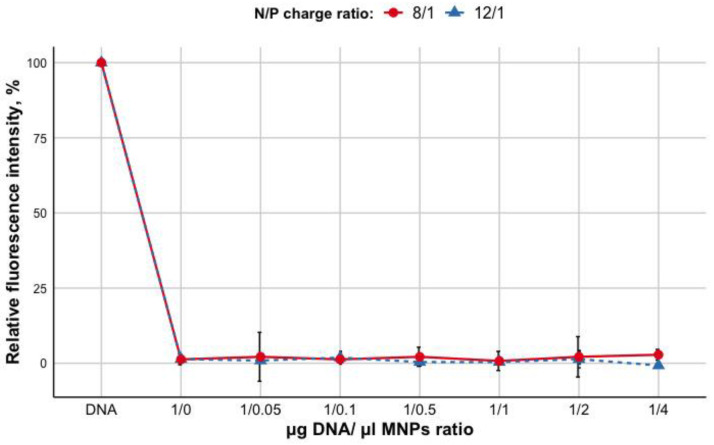
Ethidium bromide (EtBr) exclusion from R6p-cRGD/DNA polyplexes with an increasein MNPs content. Values are the mean ± S.D. of triplicate experiments.

The DNA condensation within the polyplexes with R6p-cRGD at different peptide NH_3_+/DNA PO_4−_(N/P) ratios (including 8/1 and 12/1 N/P ratios) has been reported previously [21]. To evaluate whether the addition of MNPs leads to the release of DNA from the polyplexes, ethidium bromide (EtBr) exclusion assay was used. Fluorescence of ethidium bromide bound to plasmid DNA in the absence of the carrier was taken as 100%. Different quantity ratios of R6p-cRGD/DNA and MNPs (μg DNA/μLMNPs) were tested. As shown in Figure 1, the increasing of MNPs content (0.05–4) does not lead to EtBr intercalation and an increase of fluorescence as seen at ratios of 1/0.05 to 1/4 for R6p-cRGD/DNA/MNPs. These results demonstrate that the addition of MNPs to the nucleopeptide polyplexes does not influence the nucleopeptide polyplexes density and DNA binding ability of peptide carrier R6p-cRGD.

**Figure 2:**
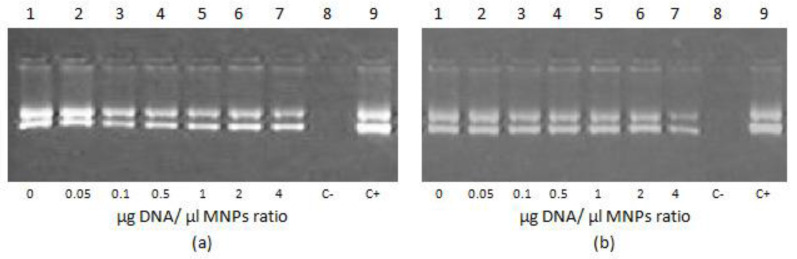
Efficiency of DNA protection from nuclease degradation within R6p-cRGD/DNA/MNPs polyplexes after DNase I enzyme digestion. The polyplexes containing 0.5 µg of DNA were treated with 0.2 units of DNase I for 30 min at 37 °C. C− is DNA treated with DNase I; C+ is intact DNA. (**a**) R6p-cRGD/DNA N/P ratio is of 8/1; (**b**) R6p-cRGD/DNA N/P ratio is of 12/1.

DNA integrity in polyplexes with R6p-cRGD and MNPs was studied using DNAse I protection assay. DNA binding by polycations is manifested in protection from nucleases [26]. As can be seen in Figure 2, the naked DNA is completely degraded by incubation with DNase I (lines 8). As was shown previously, DNA binding with R6p-cRGD remains stable after its release from polyplexes [21]. The addition of MNPs does not lead to a decrease in fluorescence for polyplexes at both N/P ratios (8/1 and 12/1, data not shown).

### 3.2. DNA Release after DTT and DS Treatment of the Polyplexes

**Figure 3:**
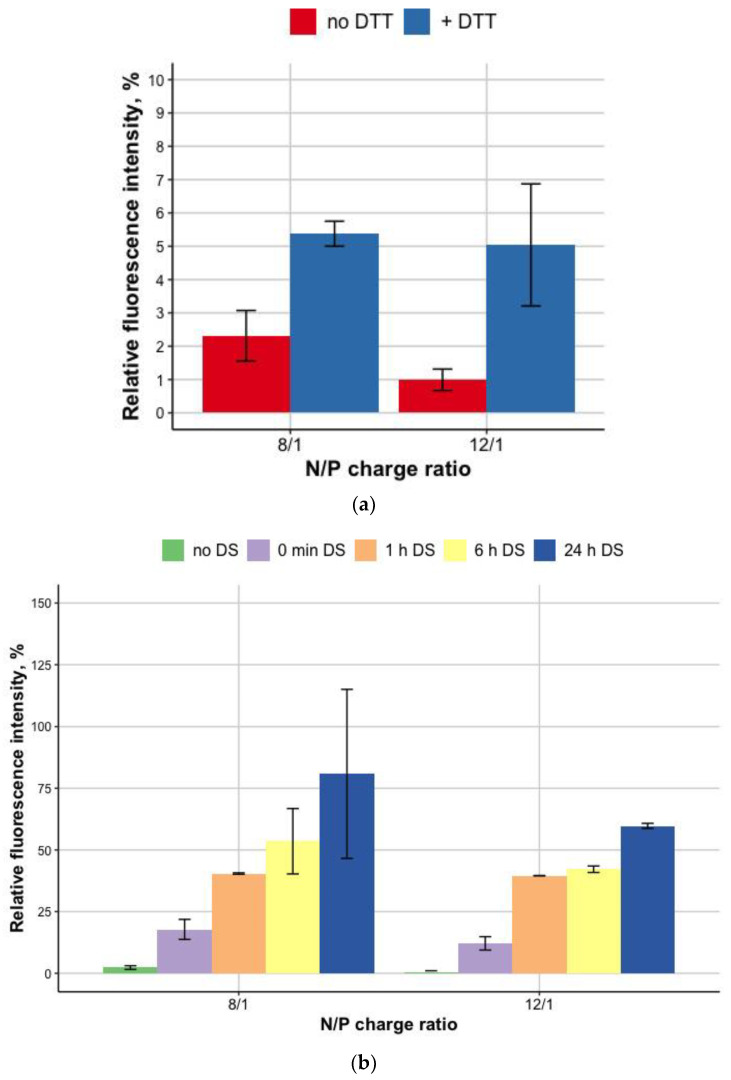
DNA release after (**a**)DTT treatment of R6p-cRGD/DNA/MNPs polyplexes; (**b**) relaxation of R6p-cRGD/DNA/MNPs polyplexes after 24h of DS treatment in three-fold charge excess. Values are the mean ± SD of the mean of triplicates.

To prove the disulphide bonds role in the formation of R6p-cRGD/DNA in MNPs-containing polyplexes, we applied dithiotreitol (DTT) as a reducible agent. DTT treatment was performed for MNPs-polyplexes at a 1:0.05 *w*/*v* ratio of DNA (μg) to MNPs (μL) (Figure 3a). We observed no destabilization of DNA condensation for all the tested MNPs-polyplexes before DTT treatment. After 1 h incubation of the MNPs-polyplexes with DTT, a fractional DNA release from polyplexes was detected. The addition of DTT resulted in a 2.5–5-fold increase in fluorescence intensity, reflecting the release of DNA from the polyplexes with MNPs. The values of the relative fluorescence after incubation with DTT compared to free DNA (100%, not shown) indicate a mild effect of disulfide bonds on the stability of polyplexes, in comparison with electrostatic forces associated with the tight DNA packaging by oligoarginine [27]. Additional experiments on the stability of MNPs-polyplexes were conducted with dextran–sulfate treatment (Figure 3b).

The interaction of polyplexes with negatively-charged GAGs (heparan sulfate, chondroitin sulfates B and C, etc.) may be tricky. On one hand, these interactions may influence the compactization of DNA in polyplexes, leading to a decrease in transfection efficiency [28]. On the contrary, DNA release from the polyplexes inside the cells is a crucial step toward transgene expression and depends on interactions between mRNAs and polyplexes [29]. In addition, the presence of some sulfated GAGs on the cell surface may also augment gene delivery [30]. Previously we showed that the R6p-cRGD/DNA polyplexes are susceptible to dextran-sulfate and easily relaxed by three-fold charge excess of DS [21]. Herein, compared to that data, the addition of MNPs does not lead to an increase in polyplexes stability (Figure 3b).

### 3.3. Size and ʐ-Potential of the Carrier/DNA/MNP Polyplexes

**Figure 4:**
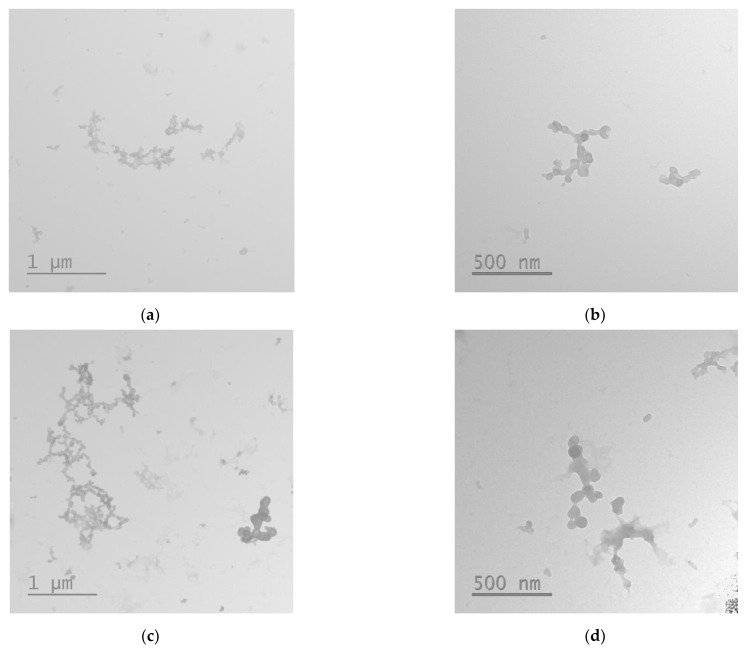
Typical micrographs of the R6p-cRGD/DNA/MNPs polyplexes obtained by transmission electron microscopy: (**a**,**b**) N/P ratio of R6p-cRGD/DNA is of 8/1; (**c**,**d**) N/P ratio is of 12/1.

One of the key features determining the internalization into the cells is the size of polyplexes. The optimal size of DNA/peptide complexes for successful transfection is considered to be <200 nm [31]. The addition of MNPs to the R6p-cRGD/DNA complexes leads to a sharp increase in size (Table 1). There are also dramatic changes in ʐ-potential of MNPs-polyplexes, which can be explained by the anionic coating of MNPs and positive charge neutralization via electrostatic interaction between R6p-cRGD/DNA complexes and MNPs. In fact, a decrease of ʐ-potential of MNPs polyplexes to nearly neutral numbers causes a decrease of the repulsion between the polyplexes and an increase of hydrodynamic size. However, according to electronic micrographs, the size of the MNPs-containing polyplexes does not correspond to data obtained using dynamic light scattering, and can be determined in the range of 80–120 nm. As can be seen in TEM images, the MNPs-containing polyplexes tend to form loosely aggregates, but in most cases have a sharp form (Figure 4).

### 3.4. Cytotoxicity Evaluation of R6p-cRGD/DNA/MNPs Polyplexes

**Figure 5:**
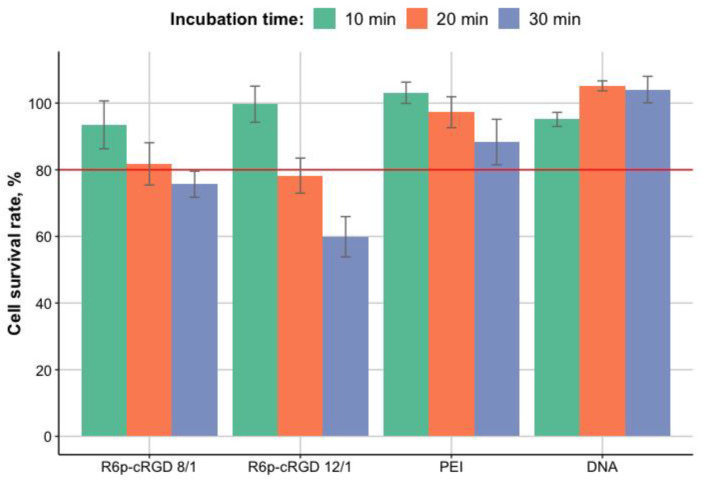
Survival rate of PANC-1 cells (%) after transfection with R6p-cRGD/DNA/MNPs polyplexes. DNA:MNP quantity ratio is 1:0.05. The threshold indicates the acceptable level of cell survival (80%).

Possible toxic effects will have a strong impact on the safety of the carrier used; thus, the absence of cytotoxicity is a crucial characteristic in carrier development. The cytotoxicity of R6p-cRGD/DNA/MNPs polyplexes to PANC-1 cells was studied using Alamar Blue (resazurin) fluorescence dye. PANC-1 cells were transfected with R6p-cRGD/DNA/MNPs polyplexes with different incubation time (10, 20, and 30 min). The fluorescence intensity of the Alamar Blue dye added to the untreated cells was taken as 100%. Naked DNA and DNA/PEI treated wells were used as a control (Figure 5).

According to previous research devoted to the cytotoxicity of PEI [32], the threshold for this experiment was set at the cell survival rate of 80%. It has been demonstrated that increasing the incubation time of R6p-cRGD/DNA/MNPs with cells leads to an increase in toxic effects. The survival rate of PANC-1 cells transfected with polyplexes with MNPs is markedly reduced, compared to control cells when the incubation time was more than 10 min (Figure 5). It has been shown that the addition of MNPs affects mostly cytotoxicity of R6p-cRGD/DNA polyplexes compared to DNA/PEI polyplexes. This can be explained by the presence of various ways of penetration of polyplexes with the developed peptide carrier into cells. Binding of the polyplexes with MNPs can promote interactions of molecules with the cell membrane [16] and the capture of more R6p-cRGD/DNA/MNPs polyplexes.

Alamar Blue assay demonstrated that the incubation of R6p-cRGD/DNA/MNPs polyplexes with cells for 10 min did not show significant cytotoxicity, indicating that this incubation time is the most optimal for successful non-toxic DNA delivery.

### 3.5. Evaluation of Magnetofection Efficacy Using R6p-cRGD/DNA/MNPs Polyplexes

**Figure 6:**
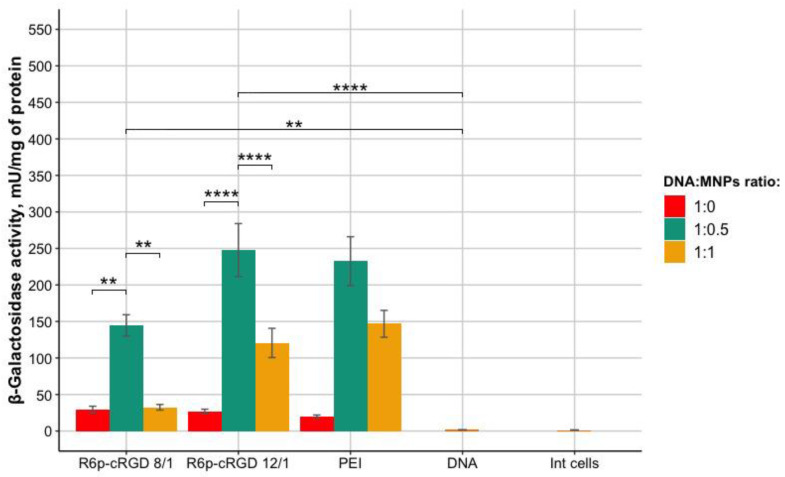
Activity of β-galactosidase in PANC-1 cells after transfection with pCMV-lacZ polyplexes with MNPs. The results are presented as mean and S.E.M. Cells transfected with R6p-cRGD/DNA/MNPs were compared to unpacked DNA; statistical significance was assessed by ordinary one-way ANOVA with Sidak’s multiple comparisons test (*n* = 9; ** 0.01, **** 0.0001).

The ability of R6p-cRGD/DNA/MNPs polyplexes to transfect cells was studied using pCMV-lacZ plasmid delivery and subsequent β-galactosidase activity evaluation. The assay was performed in PANC-1 cells.DNA packaged in a polyethyleneimine (PEI) carrier at a N/P ratio of 8/1 was used as a positive control. The efficiency of transfection was calculated by normalizing β-galactosidase activity of transfected cells to those in untreated wells. Transfection was carried out in the presence of a magnet; the incubation time of nucleopeptide polyplexes with MNPs with cells was 10 min. The results of transfection with polyplexes with the R6p-cRGD carrier and MNPs are shown in Figure 6.

Based on the results, the highest transfection efficiency was achieved using R6p-cRGD/pCMV-lacZ/MNPs polyplexes with a DNA:MNPs ratio of 1:0.5. The polyplexes without MNPs showed a low level of transfection efficiency due to the insufficient incubation time of the polyplexes with cells. In cells transfected with nucleopeptide polyplexes with a DNA:MNPs ratio of 1:1, dramatically reduced activity of the β-galactosidase enzyme was also observed in comparison with the cells transfected with polyplexes with DNA:MNPs ratio of 1:0.5. This observation can be associated with the increasing toxicity of a large number of MNPs for the PANC-1 cells.

**Figure 7:**
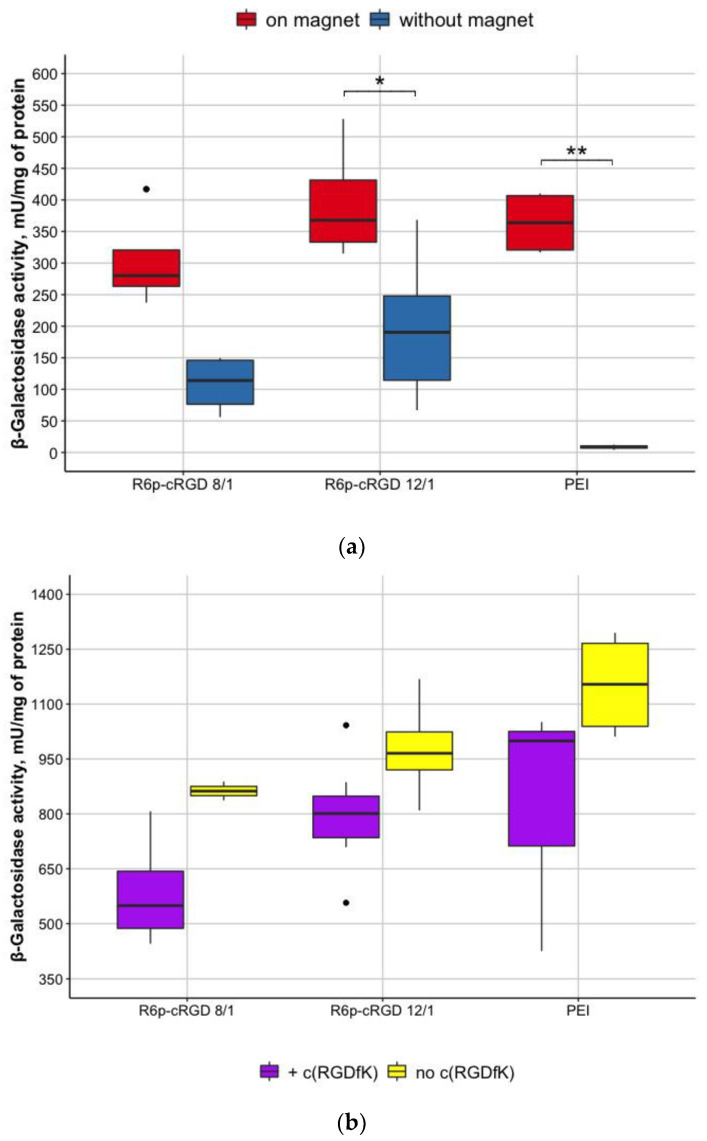
Activity of β-galactosidase after transfection of PANC-1 cells with nucleopeptide polyplexes with MNPs (**a**) in the presence and absence of a magnet; (**b**) with and without the addition of a free ligand c(RGDfK). Statistical significance was assessed by the Kruskal–Wallis test with Dunn’s multiple comparisons test (*n* = 6; * 0.05, ** 0.01).

For magnetofection efficiency evaluation, we performed transfection experiments on PANC-1 cells in the presence and absence of a magnet. We used R6p-cRGD/pCMV-lacZ polyplexes at 8/1 and 12/1 N/P ratios bound to MNPs at 1/0.5 quantity ratio of DNA:MNPs. Based on previous transfection experiments data, the incubation time of 10 min was chosen. The results demonstrated that the transfection efficacy of R6p-cRGD/pCMV-lacZ/MNPs in the presence of the magnet was significantly higher than that of the same polyplexes in the absence of the magnet (Figure 7a). Previously we demonstrated that R6p-cRGD as a carrier is able to successfully transfer DNA into the cells [21]. The decreased efficiency of polyplexes without the magnet can be explained by inappropriate incubation time of polyplexes with the cells. Besides, this testifies to the fact that MNPs themselves do not improve the transfection ability of R6p-cRGD but also do not inhibit its properties as a DNA carrier.

To evaluate whether the internalization of nucleopeptide polyplexes with MNPs occurs predominantly by receptor-mediated endocytosis due to RGD ligand in R6p-cRGD carrier, the inhibitory effect of free competing cyclic RGD (c(RGDfK)) was studied in cell transfection experiments. Competitive transfection was carried out in PANC-1 cells with R6p-cRGD/DNA polyplexes at N/P ratios of 8/1 and 12/1 and MNPs at DNA:MNPs quantitative ratio of 1/0.5in the presence of a 10-fold excess of free c(RGDfK) peptide. The magnetofection of cells with R6p-cRGD/DNA/MNPs polyplexes was held for 10 min.

The results showed a slight decrease in the efficiency of transfection in the presence of free c(RGDfK) ligand, which indicates the contribution of receptor-mediated endocytosis in the process of complex penetration into the PANC-1 cells (Figure 7b). However, despite the fact that no statistically significant differences were found, we showed that there is a tendency in decreasing in β-galactosidase activity of cells treated with c(RGDfK). The absence of a significant decrease in β-galactosidase activity of cells treated with c(RGDfK) can be explained by the presence of other internalization mechanisms. Thus, as was mentioned previously, nucleopeptide polyplexes can bind cellular membranes due to electrostatic interactions with GAGs and it can result in uptake through nonspecific endocytosis [31]. Another possible way of R6p-cRGD/DNA/MNPs internalization is macropinocytosis, that contributes to successful transfection and is not affected by the inhibiting of receptors.

### 3.6. The Therapeutic Effect of pPTK-1/R6p-cRGD/MNPs Polyplexes after Ganciclovir (GCV) Treatment

Previous research has shown that transfection with R6p-cRGD/pPTK-1 polyplexes followed by GCV treatment efficiently induces apoptosis of human UL cells [21]. To evaluate whether the use of MNPs can increase transfection efficiency of UL cells, the R6p-cRGD/pPTK-1/MNPs polyplexes were studied. We estimated the effect of suicide gene therapy by means of R6p-cRGD/pPTK-1/MNPs polyplexes after four days of GCV treatment.

**Figure 8:**
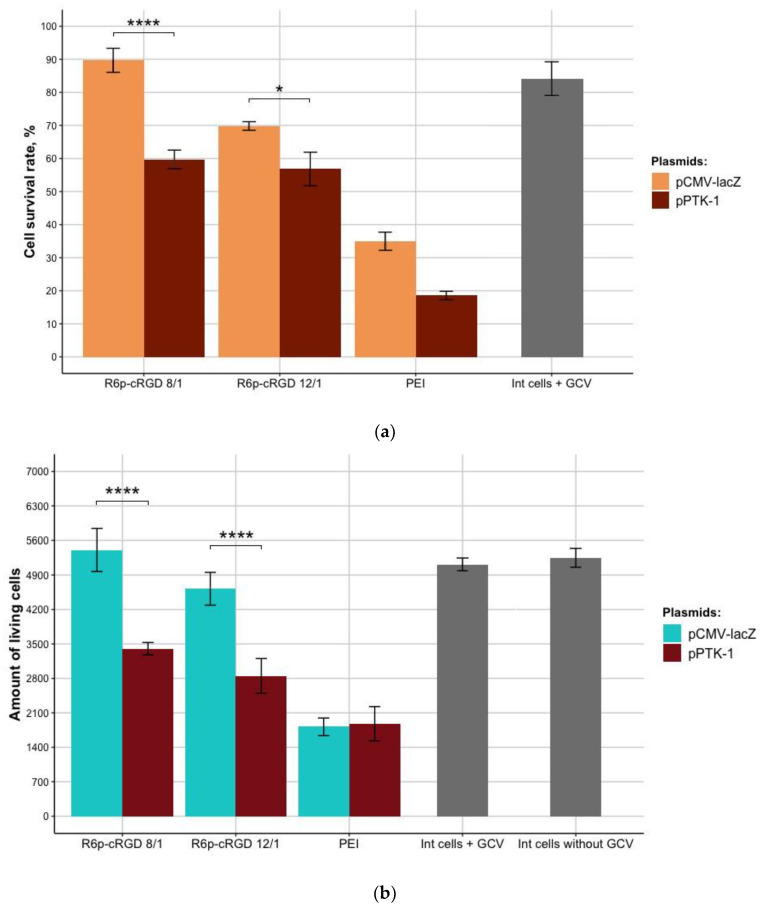
Relative number of UL cells (%) (**a**) accessed by Alamar Blue test and amount of living UL cells (**b**)accessed by Trypan blue staining after suicidal gene therapy by transfection with pPTK-1-bearing polyplexes with MNPs and subsequent incubation with ganciclovir. Statistical significance was determined using One-way ANOVA with Sidak’s multiple comparisons test (*n* = 9, * 0.05, **** 0.0001).

The cell viability after suicide gene therapy by magnetofection with R6p-cRGD/pPTK-1/MNPs (N/P 8/1) polyplexes was decreased by 33%, compared to the control pCMV-lacZ polyplexes. The UL cells proliferative activity after magnetofection with R6p-cRGD/pPTK-1/MNPs (N/P 12/1) was 1.2-fold lower, compared to R6p-cRGD/pCMV-lacZ/MNPs (Figure 8a).

The cytotoxicity caused by R6p-cRGD/pCMV-lacZ polyplexes did not exceed that of PEI-polyplexes. Moreover, PEI-polyplexes showed high cytotoxicity, and all the detected decrease in UL cell survival seems to be associated with the innate toxicity of PEI. It should be taken into account that cell survival rate of the cells transfected with PEI/pCMV-lacZ is two-fold lower than the cell survival rate for cells transfected with R6p-cRGD/pCMV-lacZ (N/P = 12/1). This observation allows us to suggest that PEI is cytotoxic for the UL cells and cannot be used as a potential carrier for gene therapy of this disease. Similar results were obtained after Trypan blue staining, allowing dead cells’ exclusion from the counting (Figure 8b). The number of living cells after transfection by pCMV-lacZ-polyplexes did not differ from that of the control cells. The number of viable UL cells transfected with R6p-cRGD/pPTK-1/MNPs polyplexes was significantly decreased up to 30%, compared to pCMV-lacZ polyplexes. Importantly, the effects of suicide gene therapy were more pronounced using Trypan blue staining rather than Alamar Blue assay.

**Figure 9:**
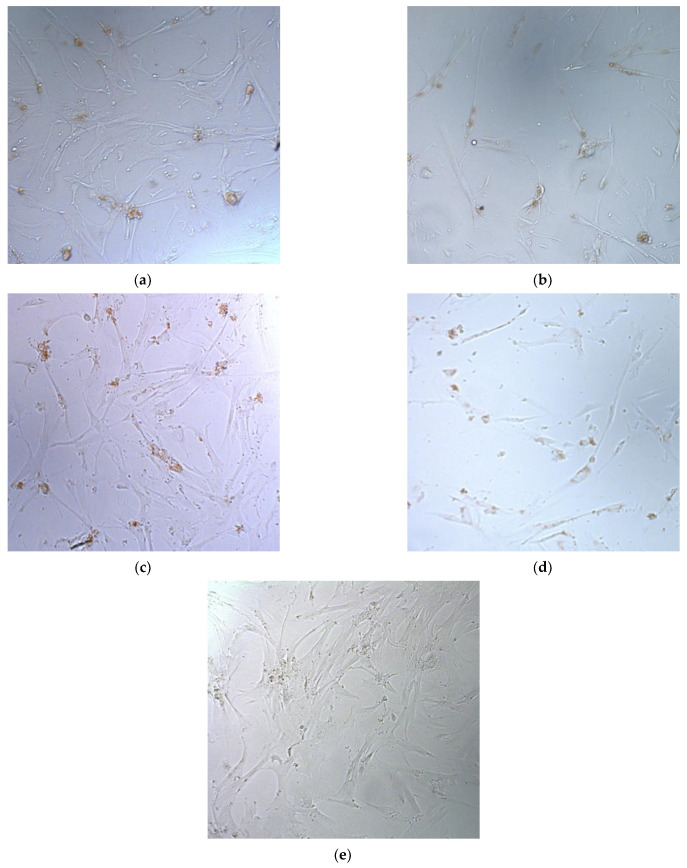
Typical micrographs in bright field conducted 96 h after GCV treatment. The UL cells were transfected with R6p-cRGD/pCMV-lacZ/MNPs polyplexes at N/P ratios of (**a**) 8/1, (**c**) 12/1; and with pPTK1/R6p-cRGD/MNPs polyplexes at (**b**) 8/1, (**d**) 12/1 charge ratio. Control wells contained (**e**) GCV-treated cells.

In addition to the Trypan blue staining test and Alamar Blue assay, micrographs of UL cells after suicide gene therapy were taken (Figure 9).

## 4. Discussion

Peptide-based carriers have demonstrated great potential for gene therapy applications in numerous in vitro and in vivo studies. They have also been used as nucleic acid delivery systems for gynaecological diseases such as endometriosis and UL [20,22,33]. Peptide carriers are non-toxic, biodegradable, and easy-to-modify polymers, making them the perfect alternative to viral vectors.

Despite various techniques applied for UL treatment, the re-appearance of tumors remains the main reason for the development of new approaches. Since gene therapy is on the upswing and the fact that precise localization makes UL a perfect target for therapeutic gene delivery in situ, this strategy began to be actively developed. In 1998, Niu and colleagues demonstrated that suicide gene therapy using non-viral vectors is applicable as a potential strategy for UL treatment [34]. After that, Salama and colleagues pointed out that the delivery of naked plasmid DNA has low efficacy in experiments in vivo. They suggested the adenoviral vector can increase the rate of delivery and successfully apply suicide gene therapy of uterine fibroids in nude mice [35]. However, the authors noted two important issues, namely the dense intercellular environment and potential cytotoxicity of gene delivery using Ad vector. These observations encouraged researchers to find a way of modification of the delivery constructs to increase the efficacy and safety of its application in vivo. Thus, one of the strategies was the binding of magnetic nanoparticles to DNA-carrying polyplexes. Magnetofection combined with adenovirus carrying herpes simplex thymidine kinase gene significantly improved the therapeutic effect after ganciclovir treatment and also allowed to decrease the viral dose and, therefore, increased safety [19].

In this work, we demonstrated that combining MNPs with R6p-cRGD complexes does not lead to their destabilization and DNA release. It allows us to consider that R6p-cRGD/DNA/MNPs polyplexes are stable at all studied quantity ratios of DNA to MNPs. However, the biocompatibility and chemical stability of MNPs are needed to be enhanced. One of the strategies to handle this is surface coating development [36].

However, despite the efficiency of magnetofection being quite high, compared with those for other non-viral carriers, its clinical use remains questionable to a certain extent. The great issue on the way to the implementation of magnetofection as an everyday tool of gene therapy is the possibility of toxic effects for the cells. After cell internalization, in late endosomes, MNPs may dissociate into ferrous ions, the size of which is small enough to let them pass through the mitochondrial membrane and be converted into ferric ions. Ferric ions can oxidize, which generates reactive oxygen species, rapid increases of which in turn can damage DNA, proteins, and lipids, resulting in cell death [37]. All of the above suggest that the optimization of magnetofection in parameters such as incubation time and MNPs quantity is of the highest priority. The short time of incubation under the action of an external magnetic field can significantly decrease undesirable side-effects related to toxicity. As we have shown in this report, incubation for 10 min promotes successful transfection without causing side-effects.

The current study showed that the addition of MNPs can also increase the efficiency of non-viral gene delivery. The results of suicide gene therapy by means of peptide carrier R6p-cRGD and MNPs are comparable with those that were shown earlier for that carrier [21]. The main improvement that was achieved using this type of modification is a 24-fold shortage in the incubation time needed for successful transfection of UL cells (from 4 h for R6p-cRGD/DNA polyplexes to 10 min for R6p-cRGD/DNA/MNPs polyplexes). Delivery of the pPTK-1 by MNPs-containing polyplexes and subsequent ganciclovir treatment resulted in a significant decrease of UL cells number, which indicates successful suicide gene therapy. This observation is confirmed by biochemical (Alamar Blue assay) and cytological (Trypan blue staining) methods and is also clearly illustrated by micrographs (Figure 9). Further improvements in R6p-cRGD polyplexes aimed at increasing stability under physiological conditions will make possible the clinical application of UL gene therapy by means of non-viral delivery. However, it is worth emphasizing the need for evaluation of these results in models in vivo. In fact, in a study by Shalaby and co-authors, the only application of magnetic force for UL gene therapy in rats is described. The authors have utilized magnetic resonance to generate a localized field in which they injected the virus into the UL tissue and restricted its spread into nearby tissues [18]. Several works devoted to magnetofection on animal models have been published recently. Another promising application of magnetic particles in gene therapy is the delivery of therapeutic constructs ex vivo. The use of magnetic nanoparticles can generally increase the efficiency of delivery into the cells [38].

## 5. Conclusions

Here, we demonstrated, for the first time, an augmentation of non-viral gene delivery to uterine leiomyoma cells using magnetic nanoparticles. Suicide gene transfer followed by ganciclovir treatment resulted in a significant increase in UL therapy. The obtained findings allow concluding that the MNPs bound to R6p-cRGD polyplexes can be considered as a promising tool for suicide gene therapy of uterine leiomyoma.

## Figures and Tables

**Table 1:** Size and ʐ-potential of the R6p-cRGD/DNA/MNPs polyplexes.

Carrier	Charge Ratio	Size (nm) ± S.D.	ʐ-Potential (mV) ± S.D.	References
R6p-cRGD	8/1	104.5 ± 0.15	32 ± 1.2	Adapted from ref. [21]
	12/1	178.4 ± 24.3	31.5 ± 0.6	
R6p-cRGD/MNPs	8/1	1814.7 ± 175.5	−5.3 ± 4.2	Current study
	12/1	1481.0 ± 182.6	4.0 ± 0.96	

## Data Availability

The data presented in this study are available on request from the corresponding author. The data are not publicly available due to restrictions of the subjects’ agreement.
